# The brain-lung immunotherapy prognostic (BLIP) Score: a novel robust tool for prognostication in non-small cell lung cancer patients with brain metastases

**DOI:** 10.1038/s41416-026-03470-6

**Published:** 2026-05-20

**Authors:** Marcus Skribek, Maria-Effrosyni Livanou, Ioannis Vathiotis, Viktor Strandman, Axel Thorell, Andreas Koulouris, Konstantinos Syrigos, Simon Ekman, Georgios Tsakonas

**Affiliations:** 1 Department of Oncology-Pathology, Karolinska Institutet, Stockholm, Sweden; 2 Medical Unit Head-Neck, Lung, Skin Cancer, Theme Cancer, Karolinska University Hospital, Stockholm, Sweden; 3 Third Department of Internal Medicine, Sotiria Thoracic Diseases Hospital of Athens, National and Kapodistrian University of Athens, Athens, Greece; 4 Laboratory of Translational Oncology, Medical School, University of Crete, Heraklion, Greece

**Keywords:** Non-small-cell lung cancer, Outcomes research

## Abstract

**Background:**

Lung cancer is the leading cause of cancer-related mortality, with brain metastases (BMs) significantly worsening prognosis. While Immune checkpoint inhibitors (ICIs) have transformed treatment for non-small cell lung cancer (NSCLC), robust prognostic tools are still lacking.

**Methods:**

The Brain-Lung Immunotherapy Prognostic (BLIP) score was developed using a retrospective cohort of NSCLC patients with BMs treated with ICIs at Karolinska University Hospital, Sweden. Prognostic factors were identified via univariate and multivariable Cox regression. Internal validation employed bootstrap resampling, penalized Cox regression and ROC analysis. External validation was conducted using an independent cohort from Sotiria Thoracic Diseases Hospital of Athens, Greece.

**Results:**

Of 1844 screened patients, 131 from Karolinska and 109 from Sotiria were included. Key variables were histology, age at BM diagnosis, and number of BMs. The BLIP score stratified patients into “Good” and “Poor” prognosis groups, with median overall survival (OS) of 14.5 and 7 months (hazard ratio [HR]: 0.4; p < 0.0001). External validation confirmed these findings (HR: 0.5; p = 0.0099).

**Conclusion:**

The BLIP score is a validated prognostic tool for NSCLC patients with BMs receiving ICIs. Incorporating clinical factors, it enhances personalized risk stratification.

**Highlights:**

- The BLIP score is a novel prognostic tool for NSCLC with brain metastases undergoing immunotherapy.
- Integrates key clinical factors like histology, age, and metastasis count.
- Internal validation demonstrates strong prognostic power and reliability.
- External validation shows effectiveness across diverse patient populations.
- Stratifies patients into “Good” and “Poor” groups, aiding in personalized treatment decisions.

## Introduction

Immune checkpoint inhibitors (ICIs), alone or in combination with chemotherapy, have demonstrated intracranial efficacy in patients with non-small cell lung cancer (NSCLC) and brain metastases (BMs) [1–5]. Nonetheless, outcomes remain heterogenous due to differences in patient and tumor characteristics, limited drug penetration across the blood–brain barrier, and resistance to prior treatments [6]. In this setting, accurate prognostic tools are essential to guide individualized care, optimize trial stratification, and inform resource allocation.

Prognostic markers estimate the likely course of disease regardless of treatment, whereas predictive markers indicate the likelihood of benefit from a specific therapy [7]. Many existing prognostic scores are based on treated populations but are not inherently predictive. Established tools such as the Recursive Partitioning Analysis (RPA) [8] and Graded Prognostic Assessment (GPA) [9, 10] rely on clinical factors but lack molecular information and do not account for ICI use. More recent models—including the disease-specific GPA (DS-GPA) [11], Lung-molGPA [12], and NSCLC GPA [6]—incorporate actionable oncogenic driver alterations and programmed death-ligand 1 (PD-L1) expression. However, PD-L1 is subject to substantial intra- and intertumoral heterogeneity, limiting its value as a purely prognostic factor and making it more suited to predicting ICI response [13–15]. Similarly, the ALK-Brain Prognostic Index (ALK-BPI) provides valuable prognostic insights but applies only to selected molecular subgroups [16]. Recent prospective studies have demonstrated improved intracranial outcomes when ICIs are combined with brain-directed radiotherapy and chemotherapy in selected patients with NSCLC and BMs, highlighting the evolving role of multimodality treatment strategies in this setting [2, 17–19].

Despite the availability of numerous models [20–25], few address the specific prognostic landscape of ICI-treated NSCLC patients with BMs. The present study aims to fill this gap by developing and externally validating the Brain-Lung Immunotherapy Prognostic (BLIP) score; an ICI-specific, clinically practical tool designed to stratify outcomes in this challenging patient population.

## Methods

### Primary patient population and study design

This retrospective cohort study included all patients with advanced NSCLC and radiologically confirmed BMs who received ICIs at Karolinska University Hospital (KUH) between July 2015 to August 2022. As the sole lung cancer care provider in the Stockholm Region, KUH manages an unselected, real-world patient population representative of routine clinical practice.

Eligible patients had BMs present at the start of ICI treatment, whether ICI was administered in the first-line or a subsequent-line setting. Patients without BMs at ICI initiation, who developed them later during ICI therapy, were not included, as intracranial outcomes could not be reliably assessed from baseline in such cases. All included patients had received at least one dose of ICI, either as monotherapy or in combination with chemotherapy. Exclusion criteria were non-NSCLC histology, absence of BMs at ICI initiation, no ICI exposure during BM presence, patients with actionable oncogenic driver alterations, or participation in clinical trials.

Data were extracted from electronic medical records. In most cases, the ICI regimen remained unchanged during the period of BM management, with any modifications based on clinical judgement. Data on baseline corticosteroid use at the time of BM diagnosis were not consistently available and were therefore not included in the analysis. Information on brain-directed radiotherapy, such as stereotactic radiosurgery or whole-brain radiotherapy, was heterogenous and often incomplete; in line with prior prognostic models, these treatment-related variables were excluded to maintain focus on baseline prognostic factors.

The study was approved by the Regional Ethical Review Board (approval no.: 2020-02636) and conducted in accordance with the Declaration of Helsinki. Informed consent was waived due to the retrospective, pseudonymized design. Treatments followed standard guidelines at the physician’s discretion, and clinical staging was based on the 8^th^ edition of the TNM classification.

### Statistical analyses and development of the BLIP score

Categorical variables were summarized descriptively, with statistical significance defined as a two-sided *p*-value < 0.05. Prognostic factors for overall survival (OS), measured from BM diagnosis, were identified using univariate and multivariable Cox proportional hazards regression, with hazard ratios (HRs) and 95% confidence intervals (95% CI). The commonly applied rule of at least 10 events per candidate variable was followed to ensure model stability, and all eligible patients were included to maximize power.

The proportional hazards assumption was tested using Schoenfeld residuals to support interpretability of HRs and overall model consistency, although not required for prognostic discrimination. All clinically relevant variables were examined in univariate analysis, and those with statistical or clinical significance were included in a multivariable model. Independent predictors were identified using backward stepwise elimination, and points were assigned according to regression coefficients and adjusted HRs (aHRs).

Candidate variables were pre-selected based on clinical relevance and statistical significance in univariate Cox regression, a standard approach used in the development of multiple prior prognostic models [8–12]. Although Akaike Information Criterion (AIC)-based model selection was also performed, yielding similar core set of prognostic variables, we prioritized interpretability and bedside utility, while addressing overfitting through penalized regression and bootstrap resampling.

Complete-case analysis was used for all included variables. Aside from PD-L1 expression, which was missing in approximately 14% in the internal cohort and 28% in the external cohort, no other key prognostic variables had missing data. No variable transformations were applied to preserve the model’s clinical simplicity and usability.

The Kaplan-Meier method was used to estimate OS by BLIP score group, with comparison performed using the log-rank test. Multiple cut-offs strategies were explored, and the cut-off with the highest prognostic discrimination was selected for the final model. Multicollinearity was assessed using the variance inflation factor (VIF). All analyses were conducted in RStudio v4.3.2 (R Foundation for Statistical Computing, Vienna, Austria).

### Outcome assessment

OS was defined as the time from BM diagnosis to death from any cause. This definition was chosen to capture prognosis following intracranial disease onset in patients treated with ICIs, while reducing heterogeneity related to treatment sequencing, timing of ICI initiation, and prior systemic therapies in a cohort receiving ICI across multiple lines of treatment. Patients alive at last follow-up were censored at the date of data cut-off.

While progression-free survival (PFS) and intracranial PFS (iPFS) may reflect short-term treatment activity, these endpoints are highly dependent on imaging schedules, assessment criteria, and post-baseline interventions. Given the retrospective, real-world nature of the cohort and the aim to develop a baseline prognostic model, PFS-based endpoints were not used for model development. Intracranial PFS was instead reported descriptively in the Supplementary Material.

### Internal validation

The BLIP score underwent internal validation using multiple statistical techniques. The dataset was randomly split into training (60%) and testing (40%) sets to ensure robustness and simulate real-world application. Kaplan-Meier curves and log-rank tests assessed survival probabilities, while Cox regression estimated HRs and 95% CIs. Although random splitting is debated in smaller datasets [26], it was applied as one of several complementary validation methods, alongside bootstrapping, Lasso penalization, and k-fold cross-validation, to assess model robustness and reproducibility.

To address overfitting and multicollinearity, Lasso penalized Cox regression was applied, with lambda (*λ*) selected via Partial Likelihood Deviance. Model stability was further tested using Efron-Gong bootstrap resampling. Ten-fold cross-validation assessed generalizability by iteratively training and validating on data subsets.

Model performance was evaluated using complementary discrimination, calibration, and clinical utility metrics. Discrimination across the entire follow-up period was assessed using the concordance index (C-index), while calibration and overall prediction accuracy were evaluated using calibration plots and the Brier score. Binary classification performance was assessed using receiver operating characteristic (ROC) curves with area under the curve (AUC), sensitivity, specificity, and Youden’s J statistic to identify optimal thresholds. Because AUC estimates depend on the time horizon of interest, time-dependent ROC analyses at 12 months were additionally performed as sensitivity analyses for both the primary and validation cohorts to assess short-term prognostic discrimination. Clinical usefulness was evaluated using decision curve analysis.

Together, these methods confirmed the score’s reliability and robustness. Further methodological details are available in the Suppl. Data.

### External validation

External validation was performed using clinical data from Sotiria Thoracic Disease Hospital of Athens (STDH), Greece, collected between December 2014 and July 2023. The same inclusion and exclusion criteria as in the KUH cohort were applied to ensure comparability. Patients were assigned BLIP scores, and survival analyses were performed using Kaplan-Meier estimates, log-rank tests, and Cox regression for hazard ratios and 95% CIs. All analyses were conducted locally under institutional ethical approval (approval no.: 7450) and in accordance with data governance protocols.

## Results

### Patient characteristics

**Fig. 1:**
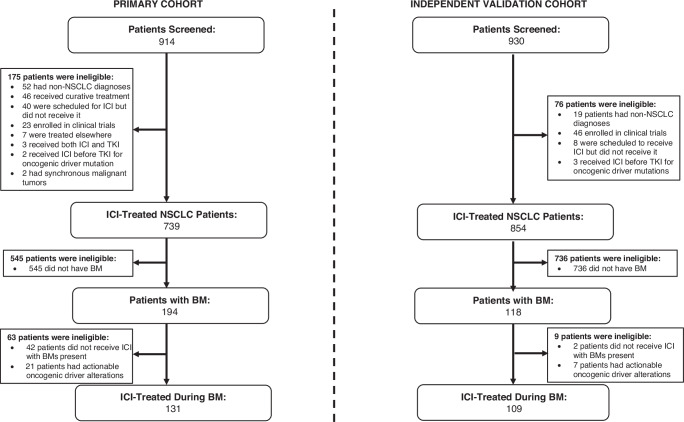
Flowchart of study design and patient population for NSCLC patients treated with ICIs across two cohorts. Flowchart of study design and patient selection for non-small cell lung cancer patients treated with immune checkpoint inhibitors in the primary and independent validation cohorts. Patients were screened and excluded based on predefined criteria, including non-NSCLC diagnoses, receipt of curative treatment, non-initiation of immune checkpoint inhibitors, enrollment in clinical trials, and presence of actionable oncogenic driver alterations. The final study population included patients treated with immune checkpoint inhibitors during the presence of brain metastases. NSCLC non-small cell lung cancer, ICI immune checkpoint inhibitor, TKI tyrosine kinase inhibitor, BM brain metastases.

Out of 914 patients screened at KUH and 930 at STDH, 131 and 109 were included, respectively (Fig. 1). Table 1 summarizes baseline patient characteristics. The primary cohort had a median follow-up of 16.0 months (58.8% female), and the validation cohort 15.0 months (78.0% male). Current smoking was more common in the validation cohort (74.3% vs. 39.7%), while non-squamous histology predominated in both (90.8% vs. 78.0%). PD-L1 > 50% was observed in 32.8% (primary) and 20.2% (validation). Median age at BM diagnosis was 68 (primary) and 65 (validation) years. ECOG PS 0–1 was seen in 81.7% vs. 67.0%, respectively, and most patients had 1–3 BMs (58.0% vs. 81.7%). MRI was more commonly used for BM diagnosis. Pembrolizumab was the most used ICI in the primary cohort (73.3%), while nivolumab dominated in the validation cohort (44.0%). Disease progression led to ICI discontinuation in most patients (74.8% vs. 78.9%). See Suppl. Table 1 for more details.

**Table 1:** Baseline patient characteristics.

Variable	Primary Cohort (*N* = 131)	Validation Cohort (*N* = 109)
**Sex**		
Male	54 (41.2%)	85 (78.0%)
Female	77 (58.8%)	24 (22.0%)
**Smoking status**		
Non-smoker	15 (11.5%)	1 (0.9%)
Former smoker	64 (48.9%)	19 (17.4%)
Current smoker	52 (39.7%)	81 (74.3%)
Missing data	0 (0.0%)	8 (7.3%)
**Histology**		
Squamous cell carcinoma	12 (9.2%)	24 (22.0%)
Non-squamous cell carcinoma	119 (90.8%)	85 (78.0%)
**PD-L1 expression**		
Low ( < 1%)	35 (26.7%)	36 (33.0%)
Intermediate (1–49%)	35 (26.7%)	20 (18.3%)
High ( ≥ 50%)	43 (32.8%)	22 (20.2%)
Not tested	18 (13.7%)	31 (28.4%)
**Primary BM**	84 (64.1%)	56 (51.4%)
**Extracranial metastasis at BM diagnosis**		
Absent	9 (6.9%)	NA
Present	122 (93.1%)	NA
**Age at BM diagnosis (years)**		
Median (IQR; range)	68 (13.5; 25–84)	65 (13; 40–82)
**ECOG PS at BM diagnosis**		
0	54 (41.2%)	17 (15.6%)
1	53 (40.5%)	56 (51.4%)
2	16 (12.2%)	16 (14.7%)
3	6 (4.6%)	7 (6.4%)
4	2 (1.5%)	3 (2.8%)
Missing data	0 (0.0%)	10 (9.2%)
**Number of BM**		
1	38 (29.0%)	54 (49.5%)
2–3	39 (29.8%)	35 (32.1%)
4–5	18 (13.7%)	12 (11.0%)
>5	33 (25.2%)	8 (7.3%)
Leptomeningeal involvement	3 (2.3%)	0 (0.0%)
**Size of largest BM (mm)**		
Median (IQR; range)	15 (17; 2–60)	13 (14; 3–47)
**Largest BM** ≥ **3 cm**		
Yes	23 (17.6%)	14 (12.8%)
Missing data	4 (3.1%)	1 (0.9%)
**Neurological symptoms associated with BM**	82 (62.6%)	39 (35.8%)
**Diagnosis of BM**		
CT	59 (45.0%)	33 (30.3%)
MRI	72 (55.0%)	76 (69.7%)
**Clinical benefit from previous therapy for BM**		
No previous treatments	54 (41.2%)	49 (45.0%)
No clinical benefit (PD)	27 (20.6%)	12 (11.0%)
Clinical benefit	17 (13.0%)	17 (15.6%)
SD	6 (4.6%)	14 (12.8%)
PR	10 (7.6%)	2 (1.8%)
CR	1 (0.8%)	1 (0.9%)
NA	33 (25.2%)	31 (28.4%)
**Age at ICI Initiation**		
Median (IQR; range)	68 (13.5; 28–84)	65 (13; 41–83)
**Regimen**		
Monotherapy	60 (45.8%)	68 (62.4%)
Combination therapy	71 (54.2%)	41 (37.6%)
**Name of ICI**		
Pembrolizumab	96 (73.3%)	43 (39.4%)
Nivolumab	24 (18.3%)	48 (44.0%)
Atezolizumab	10 (7.6%)	2 (1.8%)
Ipilimumab/Nivolumab	1 (0.8%)	16 (14.7%)
**Line of ICI therapy in metastatic lung cancer**		
1^st^ Line	73 (55.7%)	49 (45.0%)
2^nd^ Line	35 (26.7%)	43 (39.4%)
3^rd^ or Later Line	23 (17.6%)	17 (15.6%)
**Reason for therapy discontinuation**		
Disease progression	98 (74.8%)	86 (78.9%)
Toxicity	22 (16.8%)	6 (5.5%)
Non-cancer related death	4 (3.1%)	2 (1.8%)
Patient decision	3 (2.3%)	1 (0.9%)
Treatment duration over 2 years	2 (1.5%)	4 (3.7%)
Other	2 (1.5%)	5 (4.6%)
Missing Data	0 (0.0%)	5 (4.6%)
**Death**	114 (87.0%)	85 (78.0%)
**Median follow-up time**	16.0 months	15.0 months

Baseline characteristics of patients in the primary and validation cohorts. Patient demographics, disease characteristics, and treatment-related variables are presented. Continuous variables are reported as median (interquartile range [IQR] and range), and categorical variables as number (percentage).

*PD-L1* programmed death-ligand 1, *IQR* interquartile range, *ECOG PS* Eastern Cooperative Oncology Group performance status, *BM* brain metastasis, *NA* not available, *PD* progressive disease, *SD* stable disease, *PR* partial response, *CR* complete response, *ICI* immune checkpoint inhibitor.

### Clinical factors affecting the BLIP score

Univariate analysis in the primary cohort identified poorer survival with squamous histology (HR = 4.26; 95% CI, 2.3–8.0; *p* = <0.001), thoracic metastasis (HR = 1.87; 95% CI, 1.25–2.79; *p* = 0.002), age ≥65 (HR = 2.00; 95% CI, 1.34–2.98; *p* = 0.001), higher ECOG PS at BM diagnosis (HR = 2.35; 95% CI, 1.13–4.89; *p* = 0.02), >3 BMs (HR = 1.46; 95% CI, 1.00–2.13; *p* = 0.048), and age at ICI initiation (HR = 1.80; 95% CI, 1.21–2.69; *p* = 0.004) (Suppl. Table 2). In the validation cohort, thoracic metastases were not prognostic (HR = 0.78; 95% CI: 0.52–1.17; *p* = 0.24).

Multivariable analysis confirmed squamous histology (aHR = 4.60; 95% CI, 2.34–8.97; *p* < 0.001), age ≥65 (aHR = 1.73; 95% CI, 1.15–2.61; *p* = 0.009), and >3 BMs (aHR = 1.59; 95% CI, 1.06–2.36; *p* = 0.024) as independent predictors (Table 2). No multicollinearity was detected, and proportional hazards assumptions were met (Suppl. Table 3).

**Table 2:** Multivariable cox proportional hazards regression analysis of variables associated with overall survival using backward stepwise elimination.

Variables	*p*-value	aHR (95% CI)	Coefficient	VIF
Histology (squamous versus non-squamous)	<0.001***	4.593 (2.352–8.968)	1.525	1.137
Age at BM diagnosis (≥65 versus < 65)	0.009**	1.732 (1.149–2.612)	0.549	1.034
Number of BM ( > 3 versus 1–3)	0.024*	1.585 (1.064–2.363)	0.461	1.134

Multivariable Cox proportional hazards regression analysis of factors associated with overall survival. Variables included in the final model were selected using backward stepwise elimination. Adjusted hazard ratios (aHRs) with 95% confidence intervals (CIs) are presented.

*aHR* adjusted hazard ratio, *CI* confidence interval, *VIF* variance inflation factor, *BM* brain metastasis.

*P < 0.05; **P < 0.01; ***P < 0.001.

### Creation and performance of the BLIP score

Survival outcomes for prognostic factors are shown in Suppl. Table 4. Median OS was 11.0 months in the primary and 12.0 months in the validation cohort. In the primary cohort, median OS was 12.0 months (non-squamous) vs. 4.5 months (squamous); in the validation cohort, 14.0 vs. 8.0 months, respectively. Patients ≥65 years had a median OS of 9.0 months (primary) versus 11.0 months (validation). Presence of >3 BM resulted in a median OS of 9.0 months in the primary cohort versus 10.0 months in the validation cohort. Based on multivariable analysis, a point-based scoring system was created (Table 3).

**Table 3:** Calculation worksheet for the brain-lung immunotherapy prognostic (BLIP) score.

Prognostic factor	Point allocation	Patient score
**Histology**		
Non-squamous	1	
Squamous	0	
**Age at BM Diagnosis**		
<65	1	
≥65	0	
**Number of BM**		
1–3	1	
>3	0	
**Total score**	**Prognostic group**	**Median intracranial OS**
0–1	Poor	7.0 months (95% CI: 4–10)
2–3	Good	14.5 months (95% CI: 11–24)

Calculation of the Brain-Lung Immunotherapy Prognostic (BLIP) score and corresponding prognostic groups. Points are assigned based on histology, age at brain metastasis diagnosis, and number of brain metastases. Total scores classify patients into prognostic groups with corresponding median intracranial overall survival estimates.

*OS* overall survival, *CI* confidence interval, *BM* brain metastasis.

**Fig. 2:**
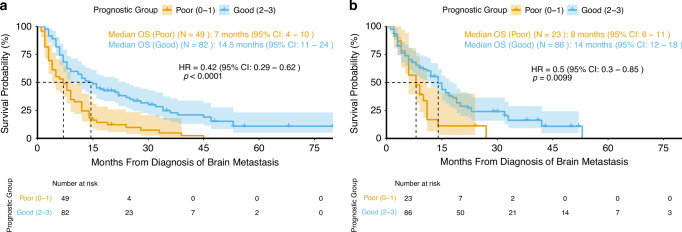
Intracranial overall survival according to BLIP prognostic group. Kaplan-Meier Survival Curves Comparing Intracranial Overall Survival in (**A**) Brain-Lung Immunotherapy Prognostic (BLIP) Groups and in the (**B**) External Validation Cohort. Kaplan–Meier curves of intracranial overall survival stratified by Brain-Lung Immunotherapy Prognostic (BLIP) score groups. **A** Survival outcomes in the primary cohort according to BLIP prognostic groups. **B** External validation of the BLIP score in an independent cohort demonstrating reproducibility of survival stratification. *OS* overall survival, *CI* confidence interval, *HR* hazard ratio.

BLIP stratified patients into “Poor” (0–1 points) and “Good” (2–3 points) groups, with median OS of 7.0 vs. 14.5 months, respectively (HR = 0.42; *p* < 0.0001) (Fig. 2A).

### Internal validation of the BLIP score

The dataset was split into training (60%) and testing (40%) sets (Suppl. Fig. 1). Both showed significant OS differences between groups (training: *p* = 0.0013; testing: *p* = 0.0017).

**Fig. 3:**
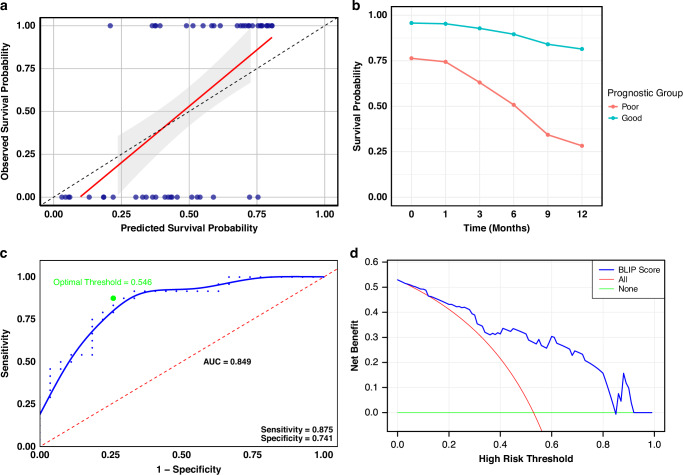
Internal validation of the brain-lung immunotherapy prognostic (BLIP) score. **A** Calibration Plot for 12-Month Survival Probabilities, **B** Predicted Survival Probabilities, **C** ROC Curve, and **D** Decision Curve Analysis. Internal validation of the brain-lung immunotherapy prognostic (BLIP) score. **A** Calibration plot for predicted versus observed 12-month survival probabilities. **B** Distribution of predicted survival probabilities. **C** Receiver operating characteristic (ROC) curve assessing discriminative ability. **D** Decision curve analysis evaluating clinical utility across a range of threshold probabilities. BLIP Brain-Lung Immunotherapy Prognostic, ROC receiver operating characteristic, AUC area under the curve.

Calibration plots confirmed alignment between predicted and observed survival (Fig. 3A), while Lasso regression showed strong calibration and performance (Suppl. Table 5). Partial likelihood deviance (Suppl. Fig. 2) remained stable for *λ* values between 4.5 and 6. Survival curves by score group showed clear separation (Fig. 3B). Model performance metrics included a Brier score of 0.16 and a c-index of 0.68, showing moderate discrimination (Suppl. Table 6). The ROC curve with an AUC of 0.85 indicated excellent discrimination (Fig. 3C). Decision curve analysis showed net benefit across thresholds (Fig. 3D). Bootstrap results are shown in Suppl. Table 7.

### External validation of the BLIP score

In the validation cohort, the “Poor” group had a median OS of 8.0 months vs. 14.0 months in the “Good” group (HR = 0.5; 95% CI: 0.30–0.85; *p* = 0.0099) (Fig. 2B), confirming the model’s prognostic utility.

### Sensitivity analysis

Ridge, Lasso, and Elastic Net regressions yielded identical AUCs (0.849). Lasso had the lowest Brier score (0.1641), indicating superior calibration and predictive accuracy (Suppl. Table 6). OS discrimination across the full follow-up period was moderate, with a C-index of 0.68. In sensitivity analyses using time-dependent ROC curves at 12 months, the BLIP score demonstrated AUCs of 0.64 (95% CI, 0.56–0.72) in the primary cohort and 0.63 (95% CI, 0.56–0.70) in the validation cohort (Suppl. Figure 3), consistent with moderate prognostic discrimination at a fixed time horizon. An extended multivariable analysis incorporating radiotherapy was also performed (Suppl. Table 8). The AIC-based model selection is reported in Suppl. Table 9 and Suppl. Table 10.

## Discussion

The BLIP score represents a novel prognostic tool for NSCLC patients with BMs receiving ICIs, integrating clinical factors to capture ICI-specific prognostic dynamics specific to the immunotherapy era. By incorporating histology, age at BM diagnosis, and number of BMs, the score stratified patients into “Poor” (0–1 points; median OS 7 months) and “Good” (2–3 points; median OS 14.5 months) prognostic groups. Discriminatory performance was confirmed by robust internal validation, and external validation in an independent cohort reinforced its reproducibility, clinical relevance, and applicability across diverse healthcare systems. While each component of the score is an established prognostic factor, their combined weighting, calibration, and external validation specifically in ICI-treated NSCLC patients with BMs is novel and addresses a clinically important gap not captured by existing models.

Traditional prognostic models, such as RPA [8] and GPA [9], predate the ICI era and lack molecular integration, limiting their utility in contemporary practice. Although DS-GPA [11] incorporates clinical features, it does not capture ICI-specific effects. More recent tools, such as Lung-molGPA [12] and NSCLC GPA [6], integrate biomarkers including PD-L1 expression; however, PD-L1 is subject to significant spatial and temporal heterogeneity, reducing its reliability as a purely prognostic factor [13–15]. While PD-L1 remains a key predictive biomarker for ICI response, its intracranial prognostic value remains uncertain, and prior studies have not demonstrated a clear association with survival in ICI treated NSCLC [27]. In line with these observations, PD-L1 expression was not independently associated with OS in our cohort and was excluded from the final model.

OS was measured from the time of BM diagnosis rather than from ICI initiation. Although OS from treatment start may appear more treatment-specific, such an approach would introduce substantial heterogeneity related to treatment sequencing, prior brain-directed therapies, and clinical selection for ICI initiation, particularly in a cohort receiving ICIs across multiple lines of therapy. Measuring OS from BM diagnosis provides a clinically meaningful and standardized prognostic anchor that reflects survival following intracranial disease onset, while preserving the baseline prognostic intent of the score. Accordingly, the BLIP score differs from existing models by focusing on ICI-treated patients and anchoring prognosis at BM diagnosis, offering a clinically relevant timeframe for contemporary practice. Variability in imaging schedules and follow-up intensity further limited reliable assessment of intracranial response, which is more appropriate for predictive rather than prognostic modeling.

Although PFS and iPFS may appear more directly linked to ICI activity, their use as primary endpoints in prognostic modeling is limited in real-world cohorts. Assessment of intracranial progression is particularly vulnerable to heterogeneity in imaging modality, follow-up intervals, lack of standardized response criteria, and informative censoring related to treatment changes or clinical deterioration. These limitations reduce the reliability and comparability of PFS-based endpoints across institutions. Accordingly, PFS and iPFS were not used for model development. Intracranial PFS was instead reported descriptively in the Supplementary Material for transparency. Importantly, PFS-based endpoints are more appropriate for predictive modeling of treatment response, whereas the BLIP score was explicitly designed as a prognostic tool to estimate survival following BM diagnosis in ICI-treated patients, independent of subsequent treatment modifications.

During the study period, targeted therapies for *KRAS* mutations were unavailable. Thoracic metastases, though significant in univariate analysis, were excluded due to uncertain independent prognostic relevance, a decision supported by external validation. Similarly, ECOG PS, did not remain independently prognostic in the multivariable model, likely reflecting correlation with age and number of BMs. Moreover, patients with poor ECOG PS are less likely to receive ICIs under standard practice, resulting in a restricted ECOG PS range that may attenuate its prognostic utility. ECOG PS would likely retain greater prognostic relevance in broader, non-ICI-selected populations.

*KRAS*-mutated disease adds further complexity, with outcomes influenced by co-mutations such as *KEAP1*, *STK11*, and *TP53* [28–30]. Recent targeted therapy trials, including CodeBreak 200, have not demonstrated an OS advantage for sotorasib over docetaxel [31], suggesting that targeted therapies may not uniformly improve survival in this setting. ICIs may therefore potentially address an unmet clinical need in mutation-positive populations, although this remains uncertain. Smoking status, a key modifier of the mutational landscape, adds another layer of prognostic complexity [32].

Recent prospective trials have demonstrated that the addition of brain-directed radiotherapy to modern systemic regimens, including ICI combined with chemotherapy, can improve intracranial disease control and survival in selected patients with NSCLC and BMs. Notably, trials such as CTONG 2003 [17], C-Brain [18], CAP-BRAIN [19], and Atezo-Brain [2] have reported favorable intracranial outcomes with upfront or concurrent radiotherapy in carefully selected populations. Importantly, these studies underscore that radiotherapy functions as a treatment-modifying intervention influenced by baseline disease burden, symptomatology, ECOG PS, access to stereotactic techniques, and institutional practice patterns. As such, radiotherapy reflects management decisions rather than intrinsic prognosis. In exploratory analyses, radiotherapy (particularly stereotactic radiosurgery) was associated with improved survival; however, this association is likely driven by selection of fitter patients with limited intracranial disease rather than a generalizable prognostic effect. Including radiotherapy in a baseline prognostic score would therefore risk conflating prognosis with treatment allocation, reduce generalizability across healthcare systems, and compromise bedside applicability. For these reasons, radiotherapy was deliberately excluded from the BLIP score, and was instead reported descriptively and in supplementary sensitivity analyses.

Baseline corticosteroid exposure could not be consistently reconstructed across both cohorts with sufficient detail regarding dose, duration, and indication. Moreover, corticosteroid use is strongly confounded by indication in routine practice [33], rendering crude binary variables difficult to interpret and prone to differential measurement error across institutions. As a pragmatic proxy for corticosteroid use, neurologic symptoms attributable to BMs were evaluated. Symptomatic CNS disease may not perfectly capture steroid exposure, albeit it was not an independently prognostic variable in our analysis.

To our knowledge, the BLIP score is the first prognostic model specifically developed for NSCLC patients with BMs treated with ICIs. By combining routinely available baseline variables, it offers a pragmatic, ICI-specific approach to outcome prediction. The use of ECOG PS instead of KPS reflects current clinical practice in the ICI era. Importantly, the BLIP score is prognostic rather than predictive, estimating survival independently of treatment response, consistent with definitions by Clark et al. [7]. A key strength is its simplicity: all variables are available at BM diagnosis, enabling immediate bedside use. To enhance usability and reduce overfitting, a simplified point-based scoring system was derived from aHRs, consistent with prior prognostic scores. Inclusion of patients treated with ICIs across multiple therapy lines further supports the robustness of real-world relevance of the score.

Internal and external validation confirmed the score’s robustness. External validation in a large, independent, and demographically distinct cohort supports its generalizability. To our knowledge, this represents the largest validation study of an ICI-specific prognostic score in NSCLC patients with BMs. Direct comparison with Lung-molGPA or NSCLC-GPA was not feasible due to fundamental differences in variable definitions, treatment eras, and biological assumptions. In particular, Lung-molGPA assigns favorable prognostic weight to *EGFR/ALK* alterations based on targeted therapy benefit, whereas such patients were excluded from our cohort. Application of these models resulted in severe subgroup fragmentation with unstable estimates, precluding meaningful comparison.

The BLIP score is intended to stratify patients by prognosis, informing monitoring intensity, palliative care integration, and trial eligibility. It is not predictive of ICI benefit and is regimen-agnostic, having been developed in patients treated with either ICI monotherapy or combination regimens. Its focus is on estimating prognosis at the time of BM diagnosis, regardless of treatment specifics. In clinical practice, “Poor” prognosis patients (BLIP score 0–1) may benefit from earlier palliative interventions, tailored surveillance, and prioritization for symptom management, while “Good” prognosis patients (BLIP score 2–3) might be considered for more aggressive multimodality approaches or clinical trial enrollment. We selected OS from the time of BM diagnosis during ICI therapy as the primary outcome to reduce confounding, given the variability in ICI initiation and BM development. This provides a more specific and clinically relevant prognostic window for ICI-treated patients with BMs.

The BLIP score demonstrated moderate discrimination across the full survival horizon (C-index 0.68), comparable to established prognostic indices in patients with BMs [34]. Discrimination varied by time horizon, with higher performance observed in binary classification analyses and more conservative estimates in time-dependent ROC analyses at 12 months. These findings highlight the complementary nature of global ranking metrics and fixed-time discrimination in heterogeneous real-world cohorts and support the BLIP score’s role as a pragmatic baseline prognostic tool rather than a high-dimensional predictive model.

This study has limitations. As a retrospective analysis, it carries inherent risks of selection bias and limited causal inference. However, including all NSCLC patients (all comers) treated with ICIs across the Stockholm Region, where KUH is the sole lung cancer provider, ensures a representative real-world cohort. The relatively small sample size increases the risk of Type II error, though this was mitigated through robust internal validation and supported by external validation in an independent cohort. Although 30% of the validation cohort lacked oncogenic testing, similar OS outcomes suggest minimal impact on overall findings. Future studies should validate these findings in larger, prospective, multicenter cohorts and explore incorporation of additional biomarkers to enhance prognostic precision.

## Conclusions

The BLIP score offers a meaningful advancement in prognostication for NSCLC patients with BMs undergoing ICI therapy, addressing key limitations of existing models. Its strong internal and external validation supports its reliability across varied clinical contexts. Despite the retrospective design and model’s sample size, the score effectively stratifies patients and holds promise for guiding personalized treatment decisions and enhancing prognostic precision.

## Supplementary information

Supplementary Data

Supplementary Material

ICMJE Form

## Data Availability

The data that support the findings in this study are available from the corresponding author, M.S., upon reasonable request.
