# Supplementary material for: The brain-lung immunotherapy prognostic (BLIP) Score: a novel robust tool for prognostication in non-small cell lung cancer patients with brain metastases
Source: Br J Cancer. 2026 May 20;135(4):621–9. doi: 10.1038/s41416-026-03470-6 (PMC13427757; doi:10.1038/s41416-026-03470-6)
Supplement: Supplementary file 2 — Supplementary Material [file 41416_2026_3470_MOESM2_ESM.docx]

| **Variable** | **Primary Cohort (N = 131)** | **Validation Cohort (N = 109)** |
| --- | --- | --- |
| **Sex** |  |  |
| Male | 54 (41.2%) | 85 (78.0%) |
| Female | 77 (58.8%) | 24 (22.0%) |
| **Smoking status** |  |  |
| Non-smoker | 15 (11.5%) | 1 (0.9%) |
| Former smoker | 64 (48.9%) | 19 (17.4%) |
| Current smoker | 52 (39.7%) | 81 (74.3%) |
| Missing data | 0 (0.0%) | 8 (7.3%) |
| **Histology** |  |  |
| Adenocarcinoma | 107 (81.7%) | 72 (66.1%) |
| Squamous cell carcinoma | 12 (9.2%) | 24 (22.0%) |
| Other | 12 (9.2%) | 13 (11.9%) |
| NSCLC NOS/poorly differentiated | 9 (6.9%) | 4 (3.7%) |
| NSC NEC | 2 (1.5%) | 0 (0.0%) |
| Adenoid cystic | 1 (0.8%) | 0 (0.0%) |
| Large cell neuroendocrine carcinoma | 0 (0.0%) | 6 (5.5%) |
| Combined histology | 0 (0.0%) | 2 (1.8%) |
| Sarcomatoid | 0 (0.0%) | 1 (0.9%) |
| **Stage at lung cancer diagnosis** |  |  |
| 1 | 3 (2.3%) | 2 (1.8%) |
| 2 | 5 (3.8%) | 5 (4.6%) |
| 3 | 12 (9.2%) | 21 (19.3%) |
| 4 | 111 (84.7%) | 81 (74.3%) |
| **Location of metastases at lung cancer diagnosis** |  |  |
| Thoracic | 50 (38.2%) | 42 (38.5%) |
| Lymph node | 105 (80.2%) | 84 (77.1%) |
| Brain | 84 (64.1%) | 54 (49.5%) |
| Liver | 22 (16.8%) | 11 (10.1%) |
| Bone | 33 (25.2%) | 22 (20.2%) |
| Adrenal gland | 18 (13.7%) | 19 (17.4%) |
| Other | 21 (16.0%) | 15 (13.8%) |
| Subcutaneous | 4 (3.1%) | 1 (0.9%) |
| Muscle | 2 (1.5%) | 2 (1.8%) |
| Renal | 5 (3.8%) | 2 (1.8%) |
| Colon | 3 (2.3%) | 3 (2.8%) |
| Pancreas | 6 (4.6%) | 0 (0.0%) |
| Ocular | 1 (0.8%) | 0 (0.0%) |
| Soft tissue | 0 (0.0%) | 5 (4.6%) |
| Spleen | 0 (0.0%) | 2 (1.8%) |
| **PD-L1 expression** |  |  |
| Low (<1%) | 35 (26.7%) | 36 (33.0%) |
| Intermediate (1–49%) | 35 (26.7%) | 20 (18.3%) |
| High (≥50%) | 43 (32.8%) | 22 (20.2%) |
| Not analyzed | 18 (13.7%) | 31 (28.4%) |
| **Genetic mutation status^1^** |  |  |
| No mutation | 61 (46.6%) | 60 (55.0%) |
| Not tested | 6 (4.6%) | 35 (32.1%) |
| Non-actionable mutations during the study period | 91 (69.5%) | 14 (12.8%) |
| *KRAS* | 49 (37.4%) | 11 (10.1%) |
| *TP53* | 8 (6.1%) | 4 (3.7%) |
| *MET* (other than exon 14 skipping mutations) | 7 (5.3%) | 0 (0.0%) |
| *PIK3CA* | 3 (2.3%) | 1 (0.9%) |
| *PTEN* | 2 (1.5%) | 0 (0.0%) |
| *BRAF* (other than V600E) | 2 (1.5%) | 0 (0.0%) |
| *HER2* | 2 (1.5%) | 1 (0.9%) |
| *CTNNB1* | 1 (0.8%) | 1 (0.9%) |
| *MYC*-amplification | 1 (0.8%) | 0 (0.0%) |
| *CEBP* | 1 (0.8%) | 0 (0.0%) |
| *ALK* exon 23 | 1 (0.8%) | 0 (0.0%) |
| *MDM4* | 1 (0.8%) | 0 (0.0%) |
| *C-KIT* | 1 (0.8%) | 0 (0.0%) |
| *MYB-NFIB* | 1 (0.8%) | 0 (0.0%) |
| *CDK4* | 1 (0.8%) | 0 (0.0%) |
| *CDK6* | 1 (0.8%) | 0 (0.0%) |
| *FOXA1* | 1 (0.8%) | 0 (0.0%) |
| *KIF5B* | 1 (0.8%) | 0 (0.0%) |
| *RET* | 1 (0.8%) | 2 (1.8%) |
| *MAP2K1* | 1 (0.8%) | 0 (0.0%) |
| *TERT* | 1 (0.8%) | 0 (0.0%) |
| *NRAS* | 1 (0.8%) | 0 (0.0%) |
| *SMARCA4* | 1 (0.8%) | 0 (0.0%) |
| *DICER1* | 1 (0.8%) | 0 (0.0%) |
| *NF1* | 1 (0.8%) | 0 (0.0%) |
| *MODY1* | 0 (0.0%) | 1 (0.9%) |
| *RB1* | 0 (0.0%) | 1 (0.9%) |
| *AKT1* | 0 (0.0%) | 1 (0.9%) |
| *NTRK2* | 0 (0.0%) | 1 (0.9%) |
| **Primary BM** | 84 (64.1%) | 56 (51.4%) |
| **Extracranial metastasis at BM diagnosis** |  |  |
| Absent | 9 (6.9%) | NA |
| Present | 122 (93.1%) | NA |
| **Age at BM diagnosis (years)** |  |  |
| Median (IQR; range) | 68 (13.5; 25–84) | 65 (13; 40–82) |
| **ECOG PS at BM diagnosis** |  |  |
| 0 | 54 (41.2%) | 17 (15.6%) |
| 1 | 53 (40.5%) | 56 (51.4%) |
| 2 | 16 (12.2%) | 16 (14.7%) |
| 3 | 6 (4.6%) | 7 (6.4%) |
| 4 | 2 (1.5%) | 3 (2.8%) |
| Missing data | 0 (0.0%) | 10 (9.2%) |
| **Number of BM** |  |  |
| 1 | 38 (29.0%) | 54 (49.5%) |
| 2–3 | 39 (29.8%) | 35 (32.1%) |
| 4–5 | 18 (13.7%) | 12 (11.0%) |
| >5 | 33 (25.2%) | 8 (7.3%) |
| Leptomeningeal involvement | 3 (2.3%) | 0 (0.0%) |
| **Size of largest BM (mm)** |  |  |
| Median (IQR; range) | 15 (17; 2–60) | 13 (14; 3–47) |
| **Largest BM ≥3 cm** |  |  |
| Yes | 23 (17.6%) | 14 (12.8%) |
| Missing data | 4 (3.1%) | 1 (0.9%) |
| **Neurological symptoms associated with BM** | 82 (62.6%) | 39 (35.8%) |
| **Diagnosis of BM** |  |  |
| CT | 59 (45.0%) | 33 (30.3%) |
| MRI | 72 (55.0%) | 76 (69.7%) |
| **Clinical benefit to previous line of therapy for BM** |  |  |
| No previous treatments | 54 (41.2%) | 49 (45.0%) |
| No clinical benefit (PD) | 27 (20.6%) | 12 (11.0%) |
| Clinical benefit | 17 (13.0%) | 17 (15.6%) |
| SD | 6 (4.6%) | 14 (12.8%) |
| PR | 10 (7.6%) | 2 (1.8%) |
| CR | 1 (0.8%) | 1 (0.9%) |
| NA | 33 (25.2%) | 31 (28.4%) |
| **Local BM Treatment ≥3 Months before ICI Treatment** |  |  |
| Yes | 48 (36.6%) | 98 (89.9%) |
| No | 83 (63.4%) | 11 (10.1%) |
| **Local Modalities to Treat BM** |  |  |
| Whole Brain Radiotherapy | 7 (5.3%) | 80 (73.4%) |
| Stereotactic Radiosurgery | 49 (37.4%) | 21 (19.3%) |
| Surgery | 10 (7.6%) | 0 (0.0%) |
| Post-Op Radiotherapy | 5 (3.8%) | 0 (0.0%) |
| Conventional Radiotherapy | 3 (2.3%) | 0 (0.0%) |
| **Radiological Intracranial Progression During ICI Treatment Period** |  |  |
| Yes | 73 (55.7%) | 32 (29.4%) |
| No | 38 (29.0%) | 76 (69.7%) |
| NA | 20 (15.3%) | 1 (0.9%) |
| **Best Radiological Response to ICI (with or without Chemotherapy) Intracranially** |  |  |
| PD | 49 (37.4%) | 21 (19.3%) |
| SD | 23 (17.6%) | 21 (19.3%) |
| PR | 31 (23.7%) | 16 (14.7%) |
| CR | 4 (3.1%) | 13 (11.9%) |
| NA | 24 (18.3%) | 38 (34.9%) |
| **Age at ICI Initiation** |  |  |
| Median (IQR; range) | 68 (13.5; 28–84) | 65 (13; 41–83) |
| **Name of ICI** |  |  |
| Pembrolizumab | 96 (73.3%) | 43 (39.4%) |
| Nivolumab | 24 (18.3%) | 48 (44.0%) |
| Atezolizumab | 10 (7.6%) | 2 (1.8%) |
| Ipilimumab/Nivolumab | 1 (0.8%) | 16 (14.7%) |
| **Regimen** |  |  |
| Monotherapy | 72 (55.0%) | 68 (62.4%) |
| Pembrolizumab/Pemetrexed/Platinum | 50 (38.2%) | 18 (16.5%) |
| Pembrolizumab/(Nab)Paclitaxel/Platinum | 4 (3.1%) | 5 (4.6%) |
| Pembrolizumab/Pemetrexed | 2 (1.5%) | 0 (0.0%) |
| Ipilimumab/Nivolumab/Platinum/Paclitaxel | 1 (0.8%) | 4 (3.7%) |
| Ipilimumab/Nivolumab/Platinum/Pemetrexed | 0 (0.0%) | 12 (11.0%) |
| Atezolizumab/(Nab)Paclitaxel/Platinum/Bevacizumab | 1 (0.8%) | 0 (0.0%) |
| Atezolizumab/Platinum/Etoposide | 1 (0.8%) | 2 (1.8%) |
| **Line of ICI therapy in metastatic lung cancer** |  |  |
| Median (IQR; range) | 1 (1; 1–6) | 2 (1; 1–5) |
| 1^st^ Line | 73 (55.7%) | 49 (45.0%) |
| 2^nd^ Line | 35 (26.7%) | 43 (39.4%) |
| 3^rd^ or Later Line | 23 (17.6%) | 17 (15.6%) |
| **Number of cycles of ICI** |  |  |
| Median (IQR; range) | 6 (7; 1–35) | 4 (7; 1–50) |
| **Duration of ICI (days)** |  |  |
| Median (IQR; range) | 91 (150; 1–785) | 70 (179; 1–802) |
| **Reason for therapy discontinuation** |  |  |
| Disease progression | 98 (74.8%) | 86 (78.9%) |
| Toxicity | 22 (16.8%) | 6 (5.5%) |
| Non-cancer related death | 4 (3.1%) | 2 (1.8%) |
| Patient decision | 3 (2.3%) | 1 (0.9%) |
| Treatment duration over 2 years | 2 (1.5%) | 4 (3.7%) |
| Other | 2 (1.5%) | 5 (4.6%) |
| Missing Data | 0 (0.0%) | 5 (4.6%) |
| **Death** | 114 (87.0%) | 85 (78.0%) |
| **Median follow-up time** | 16.0 months | 15.0 months |
| **Intracranial PFS** | 3.0 months | 6.0 months |
| **ICI-specific PFS** | 2.0 months | 2.0 months |
| **Intracranial OS** | 10.0 months | 10.0 months |
| **ICI-specific OS** | 7.0 months | 6.0 months |

**Suppl. Table 1: Detailed Patient Characteristics**

Abbreviations: NSCLC NOS: non-small cell lung cancer not otherwise specified; NSC NEC: non-small cell neuroendocrine carcinoma; PD-L1: programmed death-ligand 1; KRAS: Kirsten rat sarcoma viral oncogene homolog; TP53: tumor protein p53; PIK3CA: phosphatidylinositol-4,5-bisphosphate 3-kinase catalytic subunit alpha; PTEN: phosphatase and tensin homolog; HER2: human epidermal growth factor receptor 2; CTNNB1: catenin beta 1; MYC: MYC proto-oncogene; CEBP: CCAAT/enhancer binding protein; MDM4 proto-oncogene; C-KIT: KIT proto-oncogene; MYB-NFIB: MYB proto-oncogene, NFIB gene fusion; CDK4: cyclin-dependent kinase 4; CDK6: cyclin-dependent kinase 6; FOXA1: forkhead box a1; KIF5B: kinesin family member 5b; RET: Ret proto-oncogene; MAP2K1: mitogen-activated protein kinase 1; TERT: telomerase reverse transcriptase; NRAS: neuroblastoma RAS viral oncogene homolog; SMARCA4: SWI/SNF related, matrix associated, actin dependent regulator of chromatin, subfamily a, member 4; DICER1: dicer 1, ribonuclease III; NF1: neurofibromin 1; MODY1: Maturity-Onset Diabetes of the Young type 1; RB1: Retinoblastoma 1; AKT1: v-Akt Murine Thymoma Viral Oncogene Homolog 1; NTRAK2: Neurotrophic Receptor Tyrosine Kinase 2; RET: Rearranged during Transfection; IQR: interquartile range; ECOG PS: eastern cooperative oncology group performance status; BM: brain metastasis; NA: not available; PD: progressive disease; SD: stable disease; PR: partial response; CR: complete response; ICI: immune checkpoint inhibitor; PD-1: programmed death-1.

^1^ Mutation categories are not mutually exclusive; patients may harbor multiple alterations.

| **Variables** | **Primary Cohort (N = 131)** | |
| --- | --- | --- |
|  | ***p*-value** | **HR (95% CI)** |
| **Sex (male versus female)** | 0.950 | 1.012 (0.697–1.470) |
| **Smoking status** |  |  |
| Non-smoker |  | 1 |
| Former Smoker | 0.112 | 1.360 (0.931–1.988) |
| Current smoker | 0.715 | 0.931 (0.636–1.364) |
| **Histology (squamous versus non-squamous)** | **<0.001 ***** | 4.257 (2.270–7.984) |
| **Stage at lung cancer diagnosis** |  |  |
| 1 |  | 1 |
| 2 | 0.421 | 0.661 (0.241–1.812) |
| 3 | 0.546 | 1.203 (0.660–2.192) |
| 4 | 0.823 | 1.058 (0.645–1.736) |
| **Location of metastasis at lung cancer diagnosis** |  |  |
| No metastasis |  | 1 |
| Thoracic | **0.002 **** | 1.868 (1.253–2.786) |
| Lymph node | 0.612 | 1.126 (0.712–1.781) |
| Brain | 0.073 | 0.706 (0.482–1.033) |
| Liver | 0.141 | 1.448 (0.884–2.372) |
| Bone | 0.485 | 1.163 (0.761–1.777) |
| Adrenal gland | 0.394 | 1.278 (0.727–2.248) |
| **PD-L1 expression** |  |  |
| Low (<1%) |  | 1 |
| Intermediate (1–49%) | 0.249 | 0.784 (0.518–1.185) |
| High (≥50%) | 0.106 | 1.376 (0.934–2.028) |
| Not tested | 0.799 | 1.074 (0.622–1.852) |
| **Primary BM (no versus yes)** | 0.073 | 0.706 (0.482–1.033) |
| **Extracranial metastasis at BM diagnosis (absent versus present)** | 0.087 | 1.822 (0.917–3.621) |
| **Age at BM diagnosis (≥65 versus <65)** | **0.001 **** | 1.997 (1.338–2.982) |
| **ECOG PS at BM diagnosis (≥3 vs 0–2)** | **0.022 *** | 2.350 (1.130–4.888) |
| **Number of BM (>3 versus 1–3)** | **0.048 *** | 1.462 (1.003–2.131) |
| **Largest BM ≥3 cm (no versus yes)** | 0.710 | 1.095 (0.679–1.764) |
| **Clinical benefit to previous line of therapy for BM** |  |  |
| No previous treatment |  | 1 |
| No clinical benefit | 0.106 | 0.700 (0.455–1.079) |
| Clinical benefit | **0.028 *** | 0.207 (0.051–0.843) |
| **Local Modalities to Treat BM** |  |  |
| Whole-Brain Radiotherapy | 0.198 | 0.553 (0.224–1.364) |
| Stereotactic Radiosurgery | **<0.001 ***** | 0.469 (0.315–0.697) |
| Surgery | 0.140 | 0.577 (0.278–1.197) |
| Post-op Radiotherapy | 0.496 | 0.705 (0.258–1.928) |
| Conventional Radiotherapy | 0.801 | 0.863 (0.273–2.723) |
| **Radiotherapy (yes versus no)** | **<0.001 ***** | 0.463 (0.315–0.681) |
| **Neurological symptoms associated with BM (yes versus no)** | 0.715 | 1.074 (0.733–1.573) |
| **Clinical benefit to previous line of therapy for BM** |  |  |
| No previous treatment |  |  |
| PD | **0.015 *** | 1.803 (1.121–2.899) |
| SD | 0.400 | 1.435 (0.619–3.329) |
| PR | 0.056 | 0.467 (0.214–1.021) |
| CR | 0.961 | 0.952 (0.132–6.877) |
| **Age at ICI Initiation (≥65 versus <65)** | **0.004 **** | 1.803 (1.207–2.693) |

**Suppl. Table 2: Univariate Cox Proportional Hazards Regression Analysis of Factors Affecting Overall Survival**

Abbreviations: HR: hazard ratio; 95% CI: 95% confidence interval; PD-L1: programmed death-ligand 1; BM: brain metastasis; ECOG PS: eastern cooperative oncology group performance status; ICI: immune checkpoint inhibitor.

* = *p* < 0.05

** = *p* < 0.01

*** = *p* < 0.001

| **Variable** | **Chi-Square** | **Degrees of Freedom** | ***p*-value** |
| --- | --- | --- | --- |
| **Histology** | 0.001 | 1 | 0.97 |
| **Age at BM diagnosis** | 0.072 | 1 | 0.79 |
| **Number of BM** | 0.002 | 1 | 0.97 |
| **Global Test** | 0.076 | 3 | 0.99 |

**Suppl. Table 3: Schoenfeld Residuals Analysis**

Abbreviations: BM: brain metastasis; NA: not available.

| **Prognostic factor** | **Primary cohort (N = 131)** | **Validation cohort (N = 109)** |
| --- | --- | --- |
|  | **Median OS** | **Median OS** |
| **Whole patient population** | 11 months (95% CI: 8–14) | 12.0 months (95% CI: 9–15) |
| **Histology** |  |  |
| Non-squamous cell carcinoma | 12.0 months (95% CI: 9–16) | 14.0 months (95% CI: 11–16) |
| Squamous cell carcinoma | 4.5 months (95% CI: 2–NR) | 8.0 months (95% CI: 6–NR) |
| **Age at BM diagnosis** |  |  |
| <65 | 16.0 months (95% CI: 9–36) | 14.0 months (95% CI: 10–23) |
| ≥65 | 9.0 months (95% CI: 7–12) | 11.0 months (95% CI: 8–15) |
| **Number of BM** |  |  |
| 1–3 | 12.0 months (95% CI: 8–22) | 12.0 months (95% CI: 10–16) |
| >3 | 9.0 months (95% CI: 7–14) | 10.0 months (95% CI: 7–NR) |

**Suppl. Table 4: Survival Outcomes Associated with Prognostic Factors**

Abbreviations: OS: overall survival; 95% CI: 95% confidence interval; NR: not reached; BM: brain metastasis.

| **Variable** | **Coefficient** |
| --- | --- |
| **Histology** |  |
| Squamous cell carcinoma | 1.402 |
| **Age at BM diagnosis** |  |
| ≥65 | 0.525 |
| **Number of BM** |  |
| >3 | 0.420 |

**Suppl. Table 5: Regression Coefficients Using the Penalized Cox Regression Method**

Abbreviations: BM: brain metastasis.

| **Regularization models** | **Area under the curve** | **Brier score** |
| --- | --- | --- |
| Lasso regression | 0.849 | 0.1641 |
| Ridge regression | 0.849 | 0.1653 |
| Elastic Net regression | 0.849 | 0.1702 |
| **Performance metrics** |  | **Value** |
| C-index |  | 0.679 |
| Brier score |  | 0.164 |

**Suppl. Table 6: Comparison of Regularization Models and Performance Metrics for the Internal Validation Model**

| **Metric** | **Value** |
| --- | --- |
| Number of Bootstrap Replicates | 1,000 |
| Median Hazard Ratio | 0.42 |
| 95% Confidence Interval | 0.28–0.61 |
| Significance | Statistically significant (*p* < 0.05) |

**Suppl. Table 7: Summary of Bootstrap Analysis**

| **Multivariable Analysis** |  | |
| --- | --- | --- |
| **Variables** | **Primary Cohort (N = 131)** | |
|  | ***p*-value** | **HR (95% CI)** |
| **Histology (squamous vs non-squamous)** | **<0.001 ***** | 3.715 (1.872–7.373) |
| **Radiotherapy (yes vs no)^1^** | **0.006 **** | 0.568 (0.380–0.847) |
| **Age at BM diagnosis (≥65 vs <65)** | **0.022 *** | 1.625 (1.072–2.461) |
| **Number of BMs (>3 vs 1–3)** | 0.075 | 1.451 (0.964–2.184) |

**Suppl. Table 8: Extended Multivariable Analysis**

Abbreviations: HR: hazard ratio; 95% CI: 95% confidence interval; BM: brain metastasis.

* = *p* < 0.05

** = *p* < 0.01

*** = *p* < 0.001

^1^ Exploratory only; radiotherapy is subject to confounding by indication and selection (eligibility, oligometastatic disease, performance status) and reflects treatment decisions and access. It was defined as local brain-directed radiotherapy within 3 months of ICI initiation.

| **Variable** | **β coefficient** | **HR (95% CI)** | ***p*-value** |
| --- | --- | --- | --- |
| **Histology (squamous versus non-squamous)** | 1.30 | 3.67 (1.88–7.16) | **<0.001 ***** |
| **Thoracic metastasis (yes versus no)** | 0.37 | 1.89 (1.22–2.92) | **0.004 **** |
| **Liver metastasis (yes versus no)** | 0.59 | 1.80 (1.05–3.07) | **0.031 *** |
| **Bone metastasis (yes versus no)** | 0.36 | 1.44 (0.91–2.27) | 0.120 |
| **Extracranial metastases at BM diagnosis (absent versus present)** | 0.64 | 1.89 (0.92–3.90) | 0.083 |
| **Age at BM diagnosis (≥65 versus <65)** | 0.69 | 1.99 (1.28–3.11) | **0.002 **** |
| **Radiotherapy within 3 months prior to ICI initiation (yes versus no)** | –0.59 | 0.56 (0.37–0.84) | **0.005 **** |

**Suppl. Table 9: Cox Proportional Hazards Model Selected Using Akaike Information Criterion (AIC) for Overall Survival**

Abbreviations: HR: hazard ratio; 95% CI: 95% confidence interval; BM: brain metastasis; ICI, immune checkpoint inhibitor.

* = *p* < 0.05

** = *p* < 0.01

*** = *p* < 0.001

| **Model** | **N** | **Events** | **AIC** | **C-index** |
| --- | --- | --- | --- | --- |
| Full baseline model | 131 | 114 | 904.09 | 0.734 |
| AIC-selected model | 131 | 114 | 887.52 | 0.706 |

**Suppl. Table 10: Comparison of Akaike Information Criterion (AIC) Model Performance Metrics**

Abbreviations: AIC, Akaike Information Criterion; HR: hazard ratio; 95% CI: 95% confidence interval.


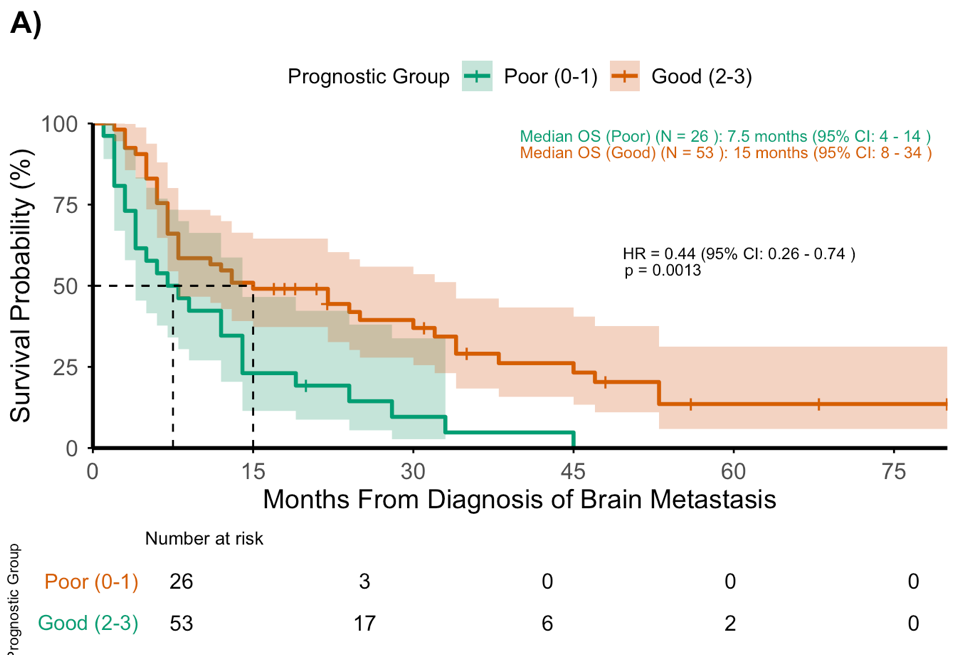

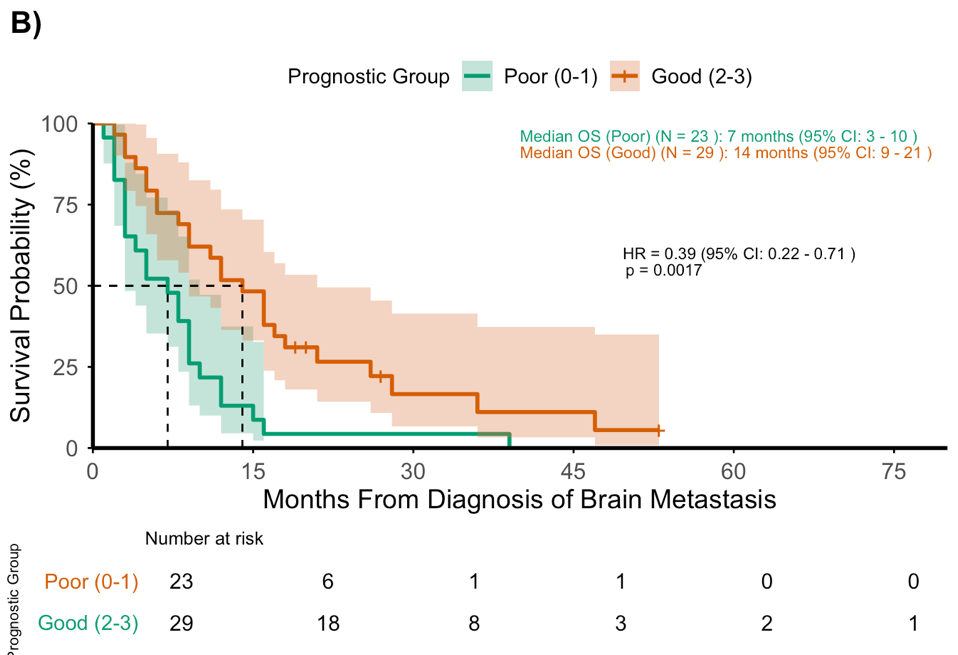


**Suppl. Figure 1: Kaplan-Meier Survival Curves for Prognostic Groups in A) the Training Cohort and B) the Testing Cohort**

Abbreviations: OS: overall survival; 95% CI: 95% confidence interval; HR: hazard ratio.


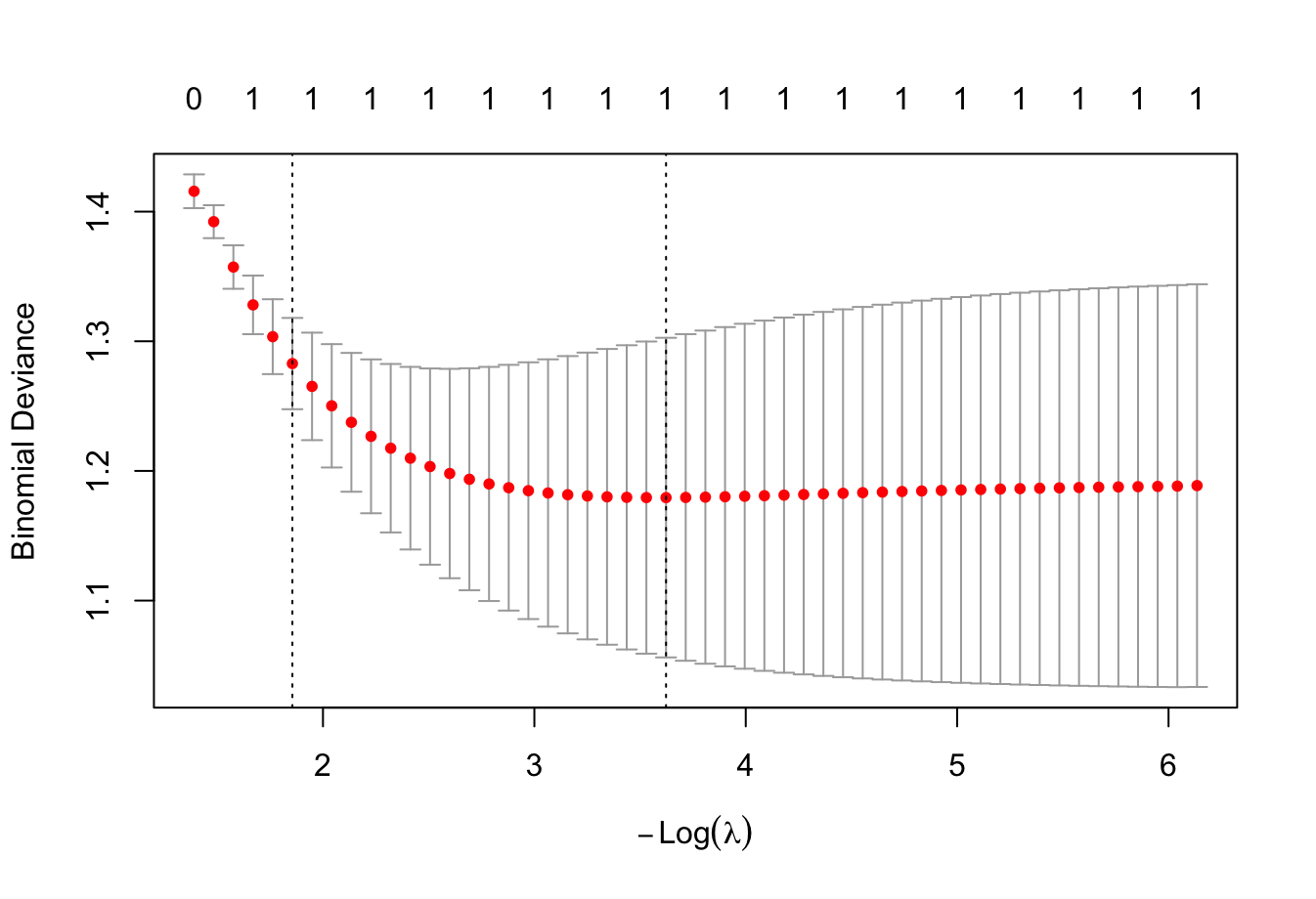


**Suppl. Figure 2: Tuning Parameter (λ) Evaluation Based on Partial Likelihood Deviance with Cross-Validation**


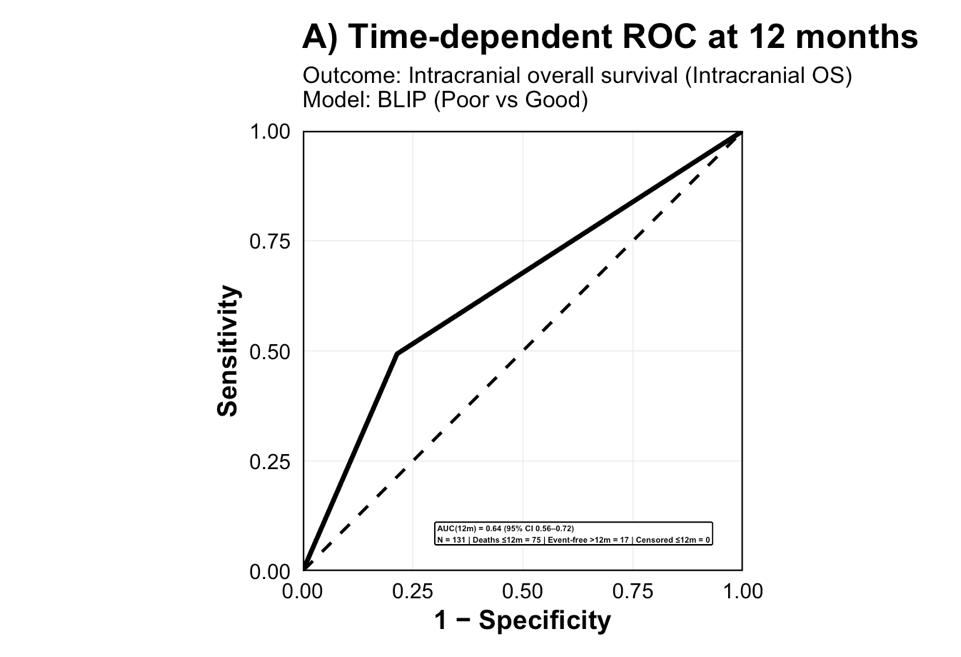

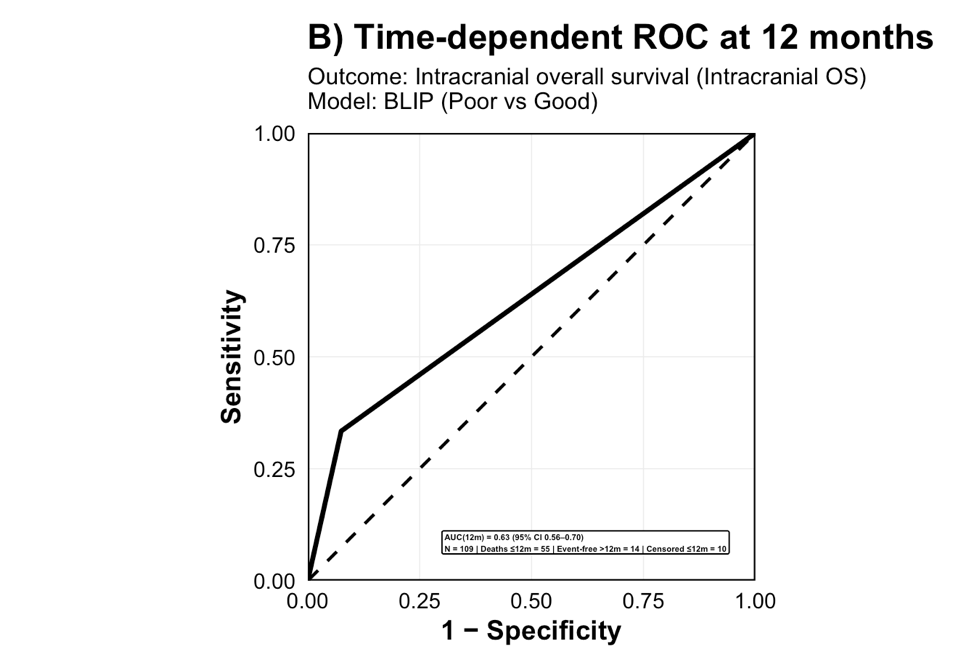


**Suppl. Figure 3: Time-dependent ROC analysis of the BLIP score at 12 months in A) The Primary Cohort and B) Validation Cohort**

Abbreviations: ROC, receiver operating characteristic; OS, overall survival; BLIP, brain-lung immunotherapy prognostic score; 95% CI, 95% confidence interval.
